# Perceptions of preclinical medical students towards extracurricular activities

**DOI:** 10.5116/ijme.5973.297a

**Published:** 2017-08-16

**Authors:** Mazen Almasry, Zeina Kayali, Rakan Alsaad, Ghada Alhayaza, Mohammad Sharique Ahmad, Akef Obeidat, Ahmed Abu-Zaid

**Affiliations:** 1 College of Medicine, Alfaisal University, Riyadh, Saudi Arabia

**Keywords:** Extracurricular activity, academic performance, medical student, curriculum, Saudi Arabia

## Abstract

**Objectives:**

To determine the percentage of students involved in extracurricular activities (EAs), explore relationships between participation in EAs and students’ characteristics, and investigate students’ perceptions (i.e., motives and barriers) towards participation in EAs.

**Methods:**

An online, anonymous, random, cross-sectional, self-rating survey was administered during spring 2015-2016 to second-year and third-year students (n=340). Chi-square test was used to explore relationships between participation in EAs and students’ characteristics. Mann-Whitney U-test was used to compare the mean 5-point Likert scale responses according to students’ characteristics. Statistical significance was determined as p<0.05.

**Results:**

Two hundred thirty-seven students participated in the survey (n=237/340, response rate: 69.7%). Only 143 students (60.3%, n=140/237) participated in EAs, and this percentage significantly differed by gender (χ^2^(1, N=237)=4.3205, p<0.037), nationality (χ^2^(1, N=237)=18.7069, p<0.000) and cumulative grade point average (cGPA, χ^2^(1, N=237)=17.8296, p<0.000). The top three motives towards participation in EAs were: “improve resume” (83.5%, n=198), “improve networking skills” (82.7%, n=196) and “improve teamwork skills” (76.8%, n=182). The top three barriers towards participation in EAs were: “lack of time” (61.2%, n=145), “lack of equal opportunities in EAs” (57.8%, n=137) and “lack of curricular emphasis of EAs” (52.7%, n=125). There was a statistically significant difference of means between male (mean=2.8) and female (mean=3.2) students regarding the following barrier: “affect academic performance negatively” (U=5389.5, p<0.002).

**Conclusions:**

The participation rate in EAs was satisfactory, and positively related to students’ characteristics of male gender, non-Saudi nationality and high cGPA. Medical schools should facilitate all potential motives and resolve all associated barriers towards participation in EAs.

## Introduction

Pre-clinical medical students spend their time mostly between curricular and extracurricular activities (EAs).^1^ Curricular activities commonly include attendance of didactic lectures, educational activities (for example, small-group teaching, problem-based learning and team-based learning) and examinations. EAs are defined as the non-academic and non-mandatory activities that are undertaken by students and fall outside the domain of the medical school curriculum.^2^ These EAs can take place inside or outside the medical school campus. Forms of EAs are diverse and include, but not limited to: research, teaching, hobbies, community service, social, cultural, religious and sports activities.^3^ For simplification purposes, EAs can be broadly dichotomized into research-related and non-research-related EAs.

Maintaining a well-judged balance between curricular and EAs is critical as it bears great impact on academic performance.^1^ Participation in research-related EAs has been linked to better academic performance and improved research-specific, higher-cognitive and transferable skills.^4^^,^^5^ Likewise, participation in non-research-related EAs, such as sports^6^^,^^7^ and service learning,^8^^,^^9^ has been associated with a superior academic performance.

Nowadays, EAs represent daily routine activities commenced by pre-clinical medical students. Furthermore, the relationship between participation in EAs and academic performance remains an endless topic of argument without reliable conclusions. The available limited literature on the topic primarily focused on the relationship between research-related EAs and academic performance.^1^^,^^10^^,^^11^ Despite students being the main stakeholders impacted by EAs, little is known about their perceptions (in terms of motives and barriers) towards participation in EAs and the relationship to academic performance. In Saudi Arabia, no such study has been conducted so far.

Corresponding to the worldwide inclination towards student-centered education,^4^ perceptions of students towards EAs offer useful information to medical educators. This information is crucial in reinforcing positive motives, correcting misconceptions, identifying barriers, employing remedial plans and reforming the medical curriculum.

This study has three aims: (1) determine the percentage of students involved in EAs, (2) explore relationships between pre-clinical students’ participation in EAs and their characteristics (gender, academic year, nationality and academic performance), and (3) investigate the students’ perceptions (in terms of motives and barriers) towards participation in EAs at Alfaisal University, College of Medicine (AU-CoM), Riyadh, Saudi Arabia. Moreover, comparisons between our study results and the relevant existing literature will be studied. The study hypothesis is that our results will be comparable to other studies reported elsewhere in the literature.

## Methods

### Study participants

At AU-CoM, the 6-year Bachelor of Medicine, Bachelor of Surgery (MBBS) program is divided into two major phases: pre-clinical (the first three years) and clinical (the last three years) phases. The targeted student participants included all pre-clinical second-year and third-year medical students (n=340). The reason for such inclusion is because these students spend the bulk of their time on campus and participate in EAs, and hence their perceptions will be beneficial and valid. The reason for excluding the first-year students was attributed to two main reasons: (1) lack of sufficient involvement in EAs during their freshman year due to the time taken to settle down and adjust to their college life, and most importantly (2) the negligible response rate received (less than 20% of the entire batch). Clinical year students were excluded from the study as their time is mostly spent in the hospitals and on clinically-related activities rather than EAs. The study protocol was approved by the Institutional Review Board (IRB) at Alfaisal University.

### Data collection method

An online, anonymous, random, non-compulsory, cross-sectional, self-rating (5-point Likert scale) study was conducted. The study took place during the spring of the 2015-2016 academic year.

The questionnaire was created based on a literature review and addition of new questions deemed significant by the authors. It was subsequently peer-reviewed by all authors and five non-author students to double-check its proper content and structure. Then, it was piloted on a convenient group of preclinical students (n=22) to inspect its validity and ensure proper interpretation of questions. Results of the piloted questions appeared to be satisfactory, appropriate and valid. Moreover, Cronbach’s α coefficient test was used to measure the extent of internal consistency among the tested items. The overall Cronbach’s α coefficient was 0.72 indicating an acceptable internal reliability of the data.^12^

At AU-CoM, the research-related EAs are largely facilitated by the student-run Undergraduate Research Committee (URC).^13^ The non-research-related EAs are largely facilitated by the student-run Medical Students’ Association (MSA). Students were asked to indicate whether they were involved in only one or both types of EAs. Moreover, students were asked to estimate the average number of hours spent on EAs per week.

Students’ characteristics data included: gender (male vs. female), academic year (second-year vs. third-year), academic performance (cumulative grade point average [cGPA] >3.0/4.0 vs. ≤3.0/4.0) and nationality (Saudi vs. non-Saudi). Academic performance was classified into: high (cGPA >3.0/4.0) and low (cGPA ≤3.0/4.0) academic performance.^11^

The students’ perceptions towards participation in EAs were evaluated by students’ responses to a total of twelve (n=12) typical 5-point Likert rating scale evaluative statements. The rating scale was as follows: (1= strongly disagree, 2= disagree, 3= neutral, 4=agree, and 5=strongly agree).

### Statistical analysis

For simplicity in reporting and analyzing data, disagreement Likert responses (1 + 2) were grouped as “disagree”; agreement responses (4 + 5) were grouped as “agree”; and neutral responses (3) were presented as “neutral”, as done previous studies.^14^^,^^15^ The average 5-point Likert scale responses were presented as means ± standard deviations (SD). All calculations of means ± SDs for all evaluative questionnaire statements were based on the 5-point Likert rating scale. Categorical data were presented as numbers and percentages. A two-tailed chi-square test of independence was used for univariate analysis (relationships) of categorical data. A two-tailed Mann-Whitney U-test was used for univariate analysis of numerical data. For all purposes, statistical significance was determined as p<0.05. All data were analyzed using the Statistical Package for Social Sciences version 24.0.

## Results

Two hundred thirty-seven students participated in the survey (n=237/340) with an overall response rate of 69.7%. Students' characteristics and their relationships to participation in EAs are illustrated in Table 1. Around 47.7% (n=113) of respondents were males and 52.3% (n=124) were females. Roughly 54% (n=128) of respondents were second-year students whereas 46% (n=109) of respondents were third-year students. There were equal percentages of Saudi and non-Saudi students (48.1% and 51.9%, respectively; n=114 and n=123, respectively). The vast majority of respondents (82.3%, n=195) had cGPA greater than 3.0/4.0. A total of 143 students (60.3%) participated in EAs, as follows: only research-related (6.3%,n=9), only non-research-related (46.2%, n=66) and both (47.5%, n=68) EAs. Moreover, almost all participating students (85.3%, n=122) reported spending less than 10 hours per week on EAs (data not reported in Table 1).

**Table 1:** Characteristics of medical students and their relationships to participation in extracurricular activities (EAs), (N=237)

Variable		Participated in EAs, N=143		Did NOT Participate in EAs, N=94	p value^*^
		n (%)		n (%)	
Gender					0.037
	Male (n=113)	76 (67.3)		37 (32.7)	
	Female (n=124)	67 (54.0)		57 (46.0)	
Academic year					0.474
	Second-year (n=128)	74 (57.8)		54 (42.2)	
	Third-year (n=109)	68 (62.4)		41 (37.6)	
Nationality					0.000
	Saudi (n=114)	52 (45.6)		62 (54.4)	
	Non-Saudi (n=123)	90 (73.2)		33 (26.8)	
Cumulative grade point average (cGPA)					
	>3.0/4.0 (n=195)		129 (66.2)	66 (33.8)	0.000
	≤3.0/4.0 (n=42)		13 (31.0)	29 (69.0)	

*Two-tailed chi-square test was used to correlate between participation in EAs and medical students’ characteristics (gender, academic year, nationality and cGPA)

Using two-tailed chi-square test (Table 1), the percentage of participation in EAs significantly differed by gender (male vs. female: 67.3% [n=76/113] vs. 32.7% [n=67/124]; χ^2^(1, N=237)=4.3205, p<0.037), nationality (Saudi vs. non-Saudi: 45.6% [n=52/114] vs. 73.2% [n=90/123]; χ^2^(1, N=237)= 18.7069, p<0.000) and cGPA (>3.0/4.0 vs. ≤3.0/4.0: 66.2% [n=129/195] vs. 33.8% [n=13/42]; χ^2^(1, N=237) = 17.8296, p<0.000).

Table 2 exhibits the students’ perceived motives towards participation in EAs. The top 3 motives were: “improve resume” (83.5%, n=198), “improve networking skills” (82.2%, n=196) and “improve teamwork skills” (76.8%, n=182).

Table 3 displays the students’ perceived barriers towards participation in EAs. The top 3 barriers were: “lack of time” (61.2%,n=145), “lack of equal opportunities in EAs” (57.8%, n=137) and “lack of emphasis of EAs in curriculum” (52.7%, n=125). Using two-tailed Mann-Whitney U-test, according to gender, there was a statistically significant difference of means between male (mean=2.8) and female (mean=3.2) students regarding the following barrier: “affect academic performance negatively” (U=5389.5, p<0.002).

**Table 2:** The perceived motives of respondents towards participation in extracurricular activities (EA), N=237

Motives for participating in EAs	All respondents^*^				Male (n=113) Mean ± SD	Female (n=124) Mean ± SD	p value**
	Disagree n (%)	Neutral n (%)	Agree n (%)	Mean ± SD			
Improving resume	13 (5.5)	26 (11.0)	198 (83.5)	4.3 ± 0.9	4.2 ± 1.0	4.3 ± 0.8	NS
Improving communication skills	17 (7.2)	44 (18.6)	176 (74.3)	4.0 ± 1.0	3.9 ± 1.0	4.1 ± 0.9	NS
Improving multi-tasking skills	15 (6.3)	54 (22.8)	168 (70.9)	3.9 ± 1.0	3.8 ± 1.1	4.1 ± 0.8	NS
Improving time management skills	22 (9.3)	60 (25.3)	155 (65.4)	3.8 ± 11	3.7 ± 1.2	4.0 ± 0.9	NS
Improving networking skills	15 (6.3)	26 (11.0)	196 (82.7)	4.2 ± 0.9	4.2 ± 1.0	4.2 ± 0.9	NS
Improving teamwork skills	17 (7.2)	38 (16.0)	182 (76.8)	4.1 ± 1.0	4.0 ± 1.1	4.1 ± 0.9	NS

*5point Likert scale: 1= strongly disagree, 2= disagree, 3= neutral, 4= agree, 5= strongly agree

**A two-tailed Mann-Whitney U-test was used to compare the mean 5-point Likert scale responses between male and female students

**Table 3:** The perceived barriers of all medical students towards participation in extracurricular activities (EA), N=237

Barriers for participating in EAs	All respondents^*^				Male (n=113) Mean ± SD	Female (n=124) Mean ± SD	p value^**^
	Disagree n (%)	Neutral n (%)	Agree n (%)	Mean ± SD			
Participation in EAs affects academic performance	56 (23.6)	116 (48.9)	65 (27.4)	3.0 ± 0.8	2.8 ± 0.8	3.2 ± 0.8	0.0466^†^
No emphasis of EAs in curriculum	32 (13.5)	80 (33.8)	125 (52.7)	3.5 ± 1.0	3.6 ± 1.1	3.5 ± 1.0	NS
No appreciation of participation in EAs	86 (36.3)	78 (32.9)	73 (30.8)	2.9 ± 1.2	2.9 ± 1.2	2.9 ± 1.2	NS
Participation in EAs is stressful	72 (30.4)	86 (36.3)	79 (33.3)	3.1 ± 1.1	3.1 ± 1.1	3.0 ± 1.1	NS
Lack of time	17 (7.2)	75 (31.6)	145 (61.2)	3.8 ± 0.9	3.8 ± 0.9	3.8 ± 0.9	NS
Lack of equal opportunities in EAs	38 (16.0)	62 (26.2)	137 (57.8)	3.7 ± 1.2	3.5 ± 1.2	3.8 ± 1.2	NS

*5-point Likert scale: 1= strongly disagree, 2= disagree, 3= neutral, 4= agree, 5= strongly agree

**A two-tailed Mann-Whitney U-test was used to compare the mean 5-point Likert scale responses between male and female students

†Statistical significance, p<0.05

## Discussion

In our study, the percentage of participation in EAs (60.3%) is reassuring and was slightly lower than another study reported elsewhere in Lebanon (76%).2 Also, the rate of spending less than 10 hours per week on EAs was similar to the average rate of 9.8 hours per week that was reported in another study conducted in the United Kingdom.^1^

There are three potential clarifications for the under-representation of female students in EAs. Firstly, female students have inclinations towards allotting greater obligations to curricular-related learning outcomes than EAs. These inclinations are plausibly reflected on their higher academic performance when compared to males.^16^^-^^18^ Secondly, female students report lower perceived proficiency in research-specific and transferable skills,^19^ both of which are needed to participate confidently in EAs. Thirdly and most importantly, the female under-representation can be related to country-specific and cultural reasons. To clarify more, education in Saudi Arabia is gender-separated in accordance with the Ministry of Higher Education regulations. Accordingly, males are largely separated from females during curricular and EAs.^15^ That being said; mixed gender interactions do occur occasionally. However, they are always regulated and mentored by medical school faculty/officials. The vast majority of EAs offered at our campus requires some interactions between both genders, and female students may refrain from participation in such EAs for self-comfort, cultural and religious reasons. Moreover, several non-research-related EAs, for example, outdoor and sports activities, are specifically logistically limited to male students.

Non-Saudi students participated more significantly in EAs than Saudi students. Generally speaking, non-Saudi graduates have small chances of being accepted into local (Saudi Arabia) and international (for example, United States of America) residency training programs. Therefore, non-Saudi students tend to have self-driven inclinations to super-compete with Saudi and American medical graduates when applying to residency training posts locally and internationally. To that end, non-Saudi students tend to work extra hard to attain higher academic performance, get engaged in scholarly research endeavors and participate in various EAs to boost their resumes and maximize their chances of acceptance.

In our study, the directly proportional relationship between cGPA and involvement in various EAs was documented in several previous studies. For instance, Lumley et al.^1^ reported that engagement in research- and teaching-related EAs was a significant factor associated with higher academic performance. Slade and Kies recently communicated a similar positive correlation between engagement in sports-based EAs and higher academic performance in final examinations.^6^ Lumley and colleagues^1^ showed that the time spent on EAs had a very negligible deleterious influence on academic performance.

Slade and Kies demonstrated that completely seizing such EAs may, in fact, negatively impact academic performance.^6^ It is our view that students with higher versus lower academic performance are more confident in their transferable skills needed to participate in a balanced curricular and EAs without incurring a negative impact on academic performance.

Improving curriculum vitae was the most frequently cited motive by students to take part in EAs. The finding was reciprocated in a previous study among medical students in the United Kingdom.^20^ Medical students may have diverse intents of engagement in EAs. While some students get engaged in EAs to shape personality and develop transferable skills, others have more strategic purposes to flourish resumes. On the other hand, some students regard EAs as respite mechanisms to cope with burnout and stress during medical school.^21^ However, irrespective of the intents, it is critical to direct students to cultivate positive attitudes towards EAs as an essential, valuable experiences to meet the demands of today’s workplace requirements.  Improving networking skills was the second most frequently cited motive to participate in EAs. This finding was echoed in an earlier study among dental students in two different Middle Eastern countries.^3^

Lack of time was the most frequently cited barrier to get engaged in EAs. Concerning research-related EAs, similar findings were demonstrated in previous reports around the world in Brazil, Canada, Kuwait, Saudi Arabia and United Kingdom.^15^^,^^19^^,^^20^^,^^22^^-^^25^

To enable smooth engagement of students in EAs, medical schools should facilitate all potential motives and resolve all associated barriers. To that end, designers of curricula should emphasize the importance of EAs and intelligently integrate research-based^26^ and non-research-based^8^^,^^9^ EAs into the curriculum. Modules of integration are many and include electives, selective and mandatory courses. Most importantly, the integration of EAs should be properly guided so that students gain the best out of their EAs for their personal development, prospective employment^27^ and stress/burnout management,^21^ however, without jeopardizing academic performance. The potential deleterious impact of EAs on academic performance can be prevented through implementation of faculty mentorship, evaluation systems and career counseling programs. Extrinsic motives such as financial rewards^28^ and academic recognition^9^ are plausible means to guarantee a continuum of participation in EAs by students.^29^

Our study has several limitations. First, the overall response rate (69.7%) was less than anticipated. Second, this is a self-reporting study conducted using an online anonymous questionnaire, and hence results were vulnerable to recall bias - that is, overestimation and underestimation of results. Moreover, the accuracy of recall information regarding academic performance and involvement in EAs were not objectively confirmed. Third, since the study design was cross-sectional, association but not causation might have been established. Causation would be more effectively demonstrated in a prospective study. Fourth, this is a single-institutional study and hence its findings cannot be generalized to reflect the status accurately in Saudi Arabia. In fact, our future research includes conducting a multi-institutional qualitative study (semi-structured focused-group interviews) to gain deeper insights into the students’ perceived motives and barriers towards participation in EAs.

## Conclusions

The participation rate in EAs was encouraging. It is vital that medical schools empower students to gain the best out of their EAs, however, without incurring unfavorable effects on academic performance. Also, lack of participation in EAs was negatively related to students’ characteristics of female gender, Saudi nationality and low academic performance (cGPA). These results highlight the importance of providing additional support to such groups of students. Lastly, the provided students’ perceptions on EAs are crucial in reinforcing positive motives, correcting misconceptions, identifying barriers and implementing solutions. 

### Conflict of Interest

The authors declare that they have no conflict of interest.

## References

[1] Lumley S, Ward P, Roberts L, Mann JP (2015). Self-reported extracurricular activity, academic success, and quality of life in UK medical students.. Int J Med Educ.

[2] Fares J, Saadeddin Z, Al Tabosh H, Aridi H, El Mouhayyar C, Koleilat MK, Chaaya M, El Asmar K (2016). Extracurricular activities associated with stress and burnout in preclinical medical students.. J Epidemiol Glob Health.

[3] Al-Ansari A, Al-Harbi F, AbdelAziz W, AbdelSalam M, El Tantawi MM, ElRefae I (2016). Factors affecting student participation in extra-curricular activities: A comparison between two Middle Eastern dental schools.. Saudi Dent J.

[4] Abu-Zaid A (2014). Research skills: the neglected competency in tomorrow's 21st-century doctors.. Perspect Med Educ.

[5] Seymour E, Hunter AB, Laursen SL, DeAntoni T (2004). Establishing the benefits of research experiences for undergraduates in the sciences: First findings from a three-year study.. Sci Ed.

[6] Slade AN, Kies SM (2015). The relationship between academic performance and recreation use among first-year medical students.. Med Educ Online.

[7] Al-Drees A, Abdulghani H, Irshad M, Baqays AA, Al-Zhrani AA, Alshammari SA, Alturki NI (2016). Physical activity and academic achievement among the medical students: A cross-sectional study.. Med Teach.

[8] Stewart T, Wubbena ZC (2015). A systematic review of service-learning in medical education: 1998-2012.. Teach Learn Med.

[9] Herlihy NS, Brown C (2015). Innovations in service learning: a novel program for community service at NYU School of Medicine.. Med Educ Online.

[10] Salgueira A, Costa P, Gonçalves M, Magalhães E, Costa MJ (2012). Individual characteristics and student's engagement in scientific research: a cross-sectional study.. BMC Med Educ.

[11] Mina S, Mostafa S, Albarqawi HT, Alnajjar A, Obeidat AS, Alkattan W, Abu-Zaid A (2016). Perceived influential factors toward participation in undergraduate research activities among medical students at Alfaisal University-College of Medicine: A Saudi Arabian perspective.. Med Teach.

[12] Tavakol M, Dennick R (2011). Making sense of Cronbach's alpha.. Int J Med Educ.

[13] Alamodi AA, Abu-Zaid A, Anwer LA, Khan TA, Shareef MA, Shamia AA, Nazmi SM, Alshammari AM, Rahmatullah H, Alsheikh AJ, Chamseddin RA, Dweik LM, Yaqinuddin A (2014). Undergraduate research: an innovative student-centered committee from the Kingdom of Saudi Arabia.. Med Teach.

[14] AlFakhri L, Sarraj J, Kherallah S, Kuhail K, Obeidat A, Abu-Zaid A (2015). Perceptions of pre-clerkship medical students and academic advisors about sleep deprivation and its relationship to academic performance: a cross-sectional perspective from Saudi Arabia.. BMC Res Notes.

[15] Kharraz R, Hamadah R, AlFawaz D, Attasi J, Obeidat AS, Alkattan W, Abu-Zaid A (2016). Perceived barriers towards participation in undergraduate research activities among medical students at Alfaisal University-College of Medicine: A Saudi Arabian perspective.. Med Teach.

[16] Lumb AB, Vail A (2004). Comparison of academic, application form and social factors in predicting early performance on the medical course.. Med Educ.

[17] Zhou YX, Zhao ZT, Li L, Wan CS, Peng CH, Yang J, Ou CQ (2014). Predictors of first-year GPA of medical students: a longitudinal study of 1285 matriculates in China.. BMC Med Educ.

[18] Salem RO, Al-Mously N, Nabil NM, Al-Zalabani AH, Al-Dhawi AF, Al-Hamdan N (2013). Academic and socio-demographic factors influencing students' performance in a new Saudi medical school.. Med Teach.

[19] Burgoyne LN, O'Flynn S, Boylan GB (2010). Undergraduate medical research: the student perspective.. Med Educ Online.

[20] Nikkar-Esfahani A, Jamjoom AA, Fitzgerald JE (2012). Extracurricular participation in research and audit by medical students: opportunities, obstacles, motivation and outcomes.. Med Teach.

[21] Fares J, Al Tabosh H, Saadeddin Z, El Mouhayyar C, Aridi H (2016). Stress, Burnout and Coping Strategies in Preclinical Medical Students.. N Am J Med Sci.

[22] de Oliveira NA, Luz MR, Saraiva RM, Alves LA (2011). Student views of research training programmes in medical schools.. Med Educ.

[23] Al-Halabi B, Marwan Y, Hasan M, Alkhadhari S (2014). Extracurricular research activities among senior medical students in Kuwait: experiences, attitudes, and barriers.. Adv Med Educ Pract.

[24] Siemens DR, Punnen S, Wong J, Kanji N (2010). A survey on the attitudes towards research in medical school.. BMC Med Educ.

[25] Russell CD, Lawson McLean A, MacGregor KE, Millar FR, Young AM, Funston GM (2012). Perceived barriers to research in undergraduate medicine.. Med Teach.

[26] Abu-Zaid A, Alkattan K (2013). Integration of scientific research training into undergraduate medical education: a reminder call.. Med Educ Online.

[27] Thompson LJ, Clark G, Walker M, Whyatt JD (2013). 'It's just like an extra string to your bow': Exploring higher education students' perceptions and experiences of extracurricular activity and employability.. Active Learning in Higher Education.

[28] Rosenkranz SK, Wang S, Hu W (2015). Motivating medical students to do research: a mixed methods study using Self-Determination Theory.. BMC Med Educ.

[29] Abu-Zaid A, BaHammam LO, Hijji TM, Shakir IM, Eshaq AM, Alawadi M, Al-Khateeb AA, Khan TA, Obeidat A, Alkattan K (2017). Extrinsic motives to encourage extracurricular research activities: a reminder call to medical schools in Saudi Arabia.. Int J Med Educ.

